# Inverting the organisational pyramid

**DOI:** 10.2349/biij.3.3.e29

**Published:** 2007-07-01

**Authors:** WR Hendee

**Affiliations:** Medical College of Wisconsin, Milwaukee, Wisconsin, United States

In any healthcare institution, the most important people are patients and their families [1]. These people benefit from the services of the institution, pay for these services either directly or indirectly through third-party payers, and hence affect the marketing of the institution through expressions of satisfaction (or dissatisfaction) with the services. They also form a customer base for receipt of present and future services from the institution.

A bond between an institution and its customer base of patients and families is essential to the survival of healthcare institutions and fulfilment of their mission as a public asset. In an organisational chart that depicts the fulfilment of an institution’s mission, patients and their families would be given “top billing” because they are the sole reason for the institution’s existence.

Within an institution, the most important employees are those who provide healthcare services to patients and supportive services to families. These persons include the nurses, technologists, orderlies, receptionists, and others who interact directly with patients and families through the provision of healthcare either directly or indirectly. These are the individuals who are fulfilling the mission of the institution, providing safe and effective healthcare services, gaining the support of patients and families for the institution, and thereby assuring the success and longevity of the institution. In cases where physicians are institutional employees, they would be included in this list of caregivers. All of these individuals should be positioned on an organisational chart directly below patients and their families because they are the conduit for the flow of services to patients and families (i.e. they are responsible for assuring the institution’s present and future customer base).

Employees who provide services to patients and families need resources and an institutional infrastructure to help them fulfil their responsibilities to patients and their families. This infrastructure includes administrative personnel who can ensure the flow of necessary resources to the employees providing services to patients. There may be multiple layers of administrative personnel. Those working most closely with the healthcare providers (the “sub-sub bosses”) constitute the top tier of administrative personnel, those who support these individuals (“the sub-bosses”) comprise the second tier, and at the bottom is the person who has the job of assuring that all of the employees above him or her have the resources and support necessary to do their job. This person is “the boss”, and his position is at the bottom of the organisational chart.

“The boss”, positioned at the bottom of an institution’s organisational chart, is responsible for assuring that the institution’s resources and infrastructure are focused appropriately to provide the needs to enable employees at the top of the chart to deliver quality healthcare services to patients and families. The boss should also serve as the institution’s principal “cheerleader” in providing an optimistic, positive atmosphere for all of the employees positioned above him or her in the organisational chart. The “boss” (and the sub- and sub-sub-bosses) set the stage for delivery of healthcare services by employees of the institution who work directly with patients and families. It is this delivery process that is fundamental to the mission and wellbeing of the institution, which is why it is positioned at the top of the organisational chart.

This understanding of the purpose of a healthcare institution and how it should function is seldom portrayed in institutional organisational charts. Instead, the charts are inverted, with “The Boss” at the top, sub-bosses below the boss, and employees who provide healthcare services at the bottom [2]. Patients and their families, who are the recipients of the institution’s services, are almost never included in the organisational chart. This portrayal reveals an introverted, self-serving way of thinking about an institution which can be (and often is) a severe handicap to the institution’s functionality and to its ability to deliver safe and effective services to patients and families. This handicap disappears when an institution recognises the need to “invert the organisational pyramid” to ensure that the delivery of services to patients and families is acknowledged as the institution’s top strategic and tactical priority.

Inversion of the organisational pyramid can (and should) occur not only in the institution as a whole, but also within every organisational subset in the institution. In a medical physics subgroup, for example, physicists working directly with physicians and technologists in the delivery of patient services are positioned above the “physics boss”, who is responsible for ensuring that those in the direct line of patient care can function optimally. Inverting the organisational pyramid does a lot to emphasise the purpose of the institution and the importance of those who are most responsible for carrying it out.
